# Re-Analyzing Differentiated High-Grade Thyroid Carcinoma and Elevated Ki-67 Proliferation: A Single-Center Retrospective Study

**DOI:** 10.3390/jcm15114173

**Published:** 2026-05-28

**Authors:** Gülsüm Karaahmetli, Şefika Burçak Polat, Sevgül Fakı, Leyla Akdoğan, Feride Pınar Altay, Birgül Genç, Ayşegül Aksoy Altınboğa, Oya Topaloğlu, Reyhan Ersoy, Bekir Çakır

**Affiliations:** 1 Department of Endocrinology and Metabolism, Ankara Bilkent City Hospital, Ankara 06800, Türkiye; black_snowtr@yahoo.com (S.F.); fpaltay@gmail.com (F.P.A.); 2 Department of Endocrinology and Metabolism, Faculty of Medicine, Ankara Yıldırım Beyazıt University, Ankara 06010, Türkiye; burcakugurlu@gmail.com (Ş.B.P.); oyasude@gmail.com (O.T.); reyhanersoy@yahoo.com.tr (R.E.); drcakir@yahoo.com (B.Ç.); 3 Department of Endocrinology and Metabolism, Kirikkale High Specialization Hospital, Kirikkale 71450, Türkiye; leyla.khrmn@hotmail.com; 4 Department of Endocrinology and Metabolism, Health Sciences Institute, Ankara Yıldırım Beyazıt University, Ankara 06800, Türkiye; bgcan2012@gmail.com; 5 Department of Pathology, Faculty of Medicine, Ankara Yıldırım Beyazıt University, Ankara 06010, Türkiye; aysegulaksoyaltinboga@aybu.edu.tr

**Keywords:** differentiated high-grade thyroid carcinoma, papillary thyroid carcinoma, Ki-67 proliferation index, mitosis, necrosis, prognosis

## Abstract

**Objective:** The 2022 WHO classification introduced differentiated high-grade thyroid carcinoma (DHGTC) as a distinct category characterized by high-grade features despite maintained differentiation. Therefore, this study aims to evaluate its clinical characteristics, treatment responses, and the prognostic impact of the Ki-67 proliferation index in this patient population. **Methods:** We retrospectively reviewed 3100 patients with differentiated thyroid carcinoma (DTC) between 2017 and 2024. From this baseline pool, a total of 56 patients (1.8%) were identified and re-classified as DHGTC based on mitotic count (≥5/2 mm^2^) and/or tumor necrosis. Additionally, 69 DTC patients (2.2%) with an elevated Ki-67 index (>5%) identified from the same baseline pool—representing an overlapping group with the DHGTC cohort—were analyzed to evaluate its clinical significance. **Results:** In the DHGTC group, tumor necrosis was present in 87.5% and high mitotic activity in 19.6% of cases. While all DHGTC patients were classified as high-risk under 2025 American Thyroid Association (ATA) guidelines, 42.8% showed biochemical or structural incomplete response at the last follow-up and 28.6% required additional salvage interventions. In the broader DTC cohort, Ki-67 ≥ 15% was significantly associated with older age, larger tumor size, extensive invasion, and poorer treatment response (*p* < 0.05). However, within the DHGTC subset, Ki-67 ≥ 15% was only significantly associated with increased lymphovascular invasion and more extensive surgery. **Conclusions:** DHGTC carries a significant burden of aggressive histopathological features and a high risk of structural disease persistence or recurrence. While an elevated Ki-67 index (≥15%) serves as an adverse marker in general DTC, its additional prognostic value within the high-grade DHGTC cohort remains inconclusive, potentially obscured by limited statistical power due to small subgroup sizes.

## 1. Introduction

The World Health Organization’s (WHO) 2022 Classification of Endocrine and Neuroendocrine Tumors introduced a distinct diagnostic category: differentiated high-grade thyroid carcinoma (DHGTC). This entity encompasses follicular-cell-derived carcinomas that exhibit high-grade features—specifically increased mitotic activity and/or tumor necrosis—while characteristically retaining the morphological and clinical features of differentiation, thereby distinguishing them from anaplastic thyroid carcinoma [1]. Under this 2022 WHO classification, DHGTC is classified alongside poorly differentiated thyroid carcinoma (PDTC) within an intermediate-prognosis group, bridging the evolutionary and prognostic gap between well-differentiated and undifferentiated thyroid malignancies [1,2].

Although DHGTC is now formally recognized, real-world data regarding its clinical behavior, long-term outcomes, and optimal management strategies remain critically limited. Much of the existing literature heavily focuses on the histopathological definition and diagnostic criteria, leaving a substantial clinical gap in understanding how these patients behave in real-world cohorts and respond to standard therapeutic modalities. Furthermore, while a high mitotic count (>5 mitoses per 2 mm^2^) and/or the presence of tumor necrosis serve as the current diagnostic gold standards, the incremental prognostic value of the Ki-67 proliferation index within this newly defined entity is not yet fully established. In routine clinical practice, Ki-67 is widely utilized as a surrogate marker of tumor aggressiveness and cellular proliferation across various endocrine tumors. However, its specific contribution to risk stratification and its ability to predict disease recurrence or treatment failure specifically within the DHGTC population require further elucidation.

To address these clinical gaps, the primary aim of this study was to characterize the clinicopathological features, treatment patterns, and long-term outcomes—including dynamic risk stratification, recurrence rates, and distant metastasis—of patients with DHGTC managed at a tertiary referral center. As a secondary objective, we investigated the clinical significance of an elevated Ki-67 index (>5%) [3,4] within a broader, comprehensive cohort of 3100 differentiated thyroid carcinoma (DTC) cases, which served as the clinical baseline for identifying high-risk proliferation. We further analyzed the DHGTC subgroup to determine whether a higher, established proliferation threshold (≥15%) [3,5]—a cutoff frequently associated with aggressive behavior and adverse therapeutic responses in endocrine malignancies—could provide additional prognostic value in predicting adverse histopathological traits and long-term treatment responses within this specific high-grade population.

## 2. Materials and Methods

### 2.1. Study Design

The study protocol was approved by the local Institutional Ethics Committee (Date: 11 March 2026; Reference No: TABED-1-26-2166) and was conducted in accordance with the ethical standards of the Declaration of Helsinki. Patients with a diagnosis of DHGTC based on total or partial thyroidectomy specimens between 2017 and 2024 were retrospectively retrieved from the pathology database by searching the keywords: necrosis, mitoses and Ki-67 proliferation index.

### 2.2. Study Population

Among the database of 3100 patients with differentiated thyroid carcinoma followed at our institution, we reviewed the histopathology reports of 2703 thyroidectomy specimens reported as DTC and included 2153 patients in which mitotic count, Ki-67 proliferation index, and the presence or absence of necrosis had been documented. From this group, 103 patients diagnosed as having PDTC were excluded from the analysis. As the term DHGTC was introduced in the 2022 WHO Classification of Endocrine and Neuroendocrine Tumors, cases diagnosed prior to this publication were reclassified as high-grade by one endocrine pathology specialist based on histopathology reports. A total of 56 patients were included in the study, either diagnosed as DHGTC after the publication of the 2022 WHO classification or reclassified as DHGTC if they fulfilled the histopathological criteria prior to 2022 (mitotic count ≥ 5 per 2 mm^2^ and/or presence of tumor necrosis).

Simultaneously, a cohort of 69 patients presenting with an elevated Ki-67 index (≥5%) was evaluated. There was a clearly defined overlap between these two study arms: 41 patients were common to both groups, representing DHGTC cases that also exhibited a Ki-67 index ≥ 5% (representing 73.2% of the DHGTC cohort). Within the remaining 15 DHGTC patients, 9 had missing Ki-67 data and 6 exhibited a Ki-67 index < 5% (their diagnosis was based purely on high mitotic count or necrosis). Conversely, 28 patients within the 69-person high Ki-67 cohort had an elevated Ki-67 index (≥5%) but did not fulfill the diagnostic criteria for DHGTC, thus remaining in the broader DTC analysis. Among the 11 DHGTC patients presenting with high mitotic activity (≥5/2 mm^2^), 8 patients concurrently exhibited a Ki-67 index ≥ 5% and were thus included within the 69-patient high Ki-67 cohort, whereas the remaining 3 patients were excluded from this cohort due to either a Ki-67 index < 5% (*n* = 1) or missing Ki-67 data (*n* = 2). The patient screening and inclusion process is summarized in Figure S1.

### 2.3. Study Protocol

To prevent any potential clinical ambiguity for the readership regarding these dual markers, it is critical to delineate the fundamental biological and methodological differences between these two proliferative assessments. Mitotic count is a purely morphological evaluation performed via light microscopy that quantifies cells captured when actively undergoing the physical phase of cellular division (mitosis per 2 mm^2^). In contrast, the Ki-67 proliferation index is an immunohistochemical marker that measures the total growth fraction of the tumor. Ki-67 protein is expressed during all active phases of the cell cycle (G_1_, S ve G_2_ and mitosis) but is strictly absent in resting cells (G_0_) Therefore, while mitotic count reflects immediate physical division at a single point in time, the Ki-67 index provides a broader estimation of the overall cellular replication potential.

Although the Ki-67 proliferation index is not classified among the high-grade features, we also examined whether a value exceeding 15% influences clinical outcomes regardless of high-grade tumor characteristics. In addition to that, the DHGTC cohort was further stratified into subgroups with Ki-67 ≥ 15% and 5–15% to evaluate its impact on tumor characteristics and outcome. The threshold values of >5% and ≥15% for the Ki-67 proliferation index were selected a priori based on previously validated clinical and prognostic cut-offs in the recent high-grade follicular cell-derived thyroid carcinoma literature [3,5]. These specific cut-offs were utilized rather than deriving an independent threshold through internal institutional ROC curve analysis, thereby avoiding statistical overfitting given our specific subgroup sample sizes.

Outcome data included the demographic data of the patients, preoperative ultrasonography (US) risk scoring according to the Thyroid Imaging Reporting and Data System (TI-RADS), fine-needle aspiration biopsy (FNAB), Bethesda category, surgery details, tumor size, initial stage, presence of local, regional, or distant recurrence and dynamic risk classification at the last visit and history of radioactive iodine (RAI) and dosage. Local or regional recurrence after treatment was determined based on cytological or histopathological confirmation of structurally identifiable disease. Local recurrence was defined as disease reappearance in the thyroid bed, confirmed by cytology or histology following additional surgery. Regional recurrence was defined as disease detected in the cervical lymph nodes, again confirmed by cytological or histopathological analysis after subsequent surgical intervention. Distant recurrence was assessed using imaging studies, including radioiodine uptake scans, computed tomography (CT), positron emission tomography (PET) scans, or, where available, cytological and histopathological evidence. Changes in thyroglobulin (Tg) levels in anti-Tg negative patients were also considered indicative of recurrence. In recent years, patients with previously undetectable Tg (<0.2 ng/mL) who develop detectable levels have been evaluated using ultrasound, fine-needle aspiration of suspicious nodes or nodules, and CT scans of the neck and chest to identify structural disease. The follow-up period for each patient was defined as the duration from the initial therapy to the last documented contact, based on a review of the medical record. Postsurgical physical examinations were carried out every 3–6 months. At each follow-up visit, all patients underwent neck ultrasonography and thyroglobulin measurement.

Treatment responses were evaluated at the last follow-up according to the American Thyroid Association (ATA) dynamic risk stratification system. Biochemical incomplete response was defined as persistent or rising thyroglobulin (Tg) or anti-thyroglobulin antibody (TgAb) levels without structural evidence of disease. Structural incomplete response was defined as the presence of biopsy-proven or radiologically identifiable persistent or recurrent locoregional or distant metastases.

All analyses were performed using SPSS version 17 (SPSS Inc., Chicago, IL, USA). Continuous variables were presented as median (range), and categorical variables were presented as counts and percentages. The normality of continuous variables was formally evaluated using the Shapiro–Wilk test alongside visual inspection of histograms. Because the primary clinical and histopathological continuous variables (including patient age, tumor size, and mitotic count) deviated significantly from a normal distribution (*p* < 0.05), non-parametric alternatives—specifically the Mann–Whitney U test—were justified and applied for assessing differences between two independent groups. Associations between categorical variables in two independent groups were evaluated using the Chi-square test or Fisher’s exact test when expected counts were low. Furthermore, to address the potential inflation of Type I error arising from the large number of concurrent variables analyzed, a Bonferroni correction was applied where appropriate for multiple comparisons, and adjusted significance thresholds were utilized. A two-sided *p*-value < 0.05 (or the corresponding Bonferroni-adjusted threshold) was considered statistically significant.

## 3. Results

**DHGTC Group demographic, clinical, histopathological and follow up**

The DHGTC cohort comprised 56 patients with a balanced gender distribution. The patient screening, definitive cohort inclusion criteria, and baseline clinical monitoring timelines were structured to evaluate long-term oncologic characteristics.

Histopathologically, aggressive features were highly prevalent, characterized by frequent capsular invasion (67.9%), lymphovascular invasion (51.8%), and positive surgical margins (39.3%). Notably, tumor necrosis was the most dominant high-grade defining feature, identified in the vast majority of patients, whereas a relatively smaller proportion of cases fulfilled the high mitotic activity (≥5/2 mm^2^) criteria alone.

A striking shift was observed when evaluating recurrence risk models; while under the historical 2015 ATA risk classification most patients were categorized as intermediate-risk and nearly one-fifth as low-risk, the updated 2025 ATA guidelines reclassified the entire cohort (100%) into the high-risk category. According to the American Joint Committee on Cancer (AJCC) 8th edition staging, the distribution was relatively balanced across all stages, with nearly half of the patients presenting with advanced stage IV disease.

In terms of preoperative ultrasonographic features evaluated prior to definitive surgical intervention and histological confirmation, the vast majority of lesions presented with higher-suspicion categories, specifically TI-RADS 4 and 5. Notably, no cases within this high-grade cohort were classified as low-risk TI-RADS 1 (Table 1).

Regarding definitive surgical management, bilateral total thyroidectomy (BTT) was the predominant operative procedure (53.6%), with more than a third of the cohort requiring concurrent central and/or lateral neck dissections (LND; 35.7%).

Adjuvant management was comprehensive, with the overwhelming majority of patients receiving radioactive iodine (RAI) therapy (91.1%). Reflecting a high risk of disease persistence and recurrence, additional therapeutic interventions—primarily reoperations, followed by radiotherapy, tyrosine kinase inhibitors, and repeated RAI courses—were required in more than a quarter of the cohort (28.6%).

At the final follow-up, dynamic risk stratification demonstrated a diverse spectrum of clinical outcomes; while roughly one-third of the cohort achieved an excellent biochemical response (30.4%), a substantial proportion of patients exhibited a structural incomplete response (35.7%), radiologically confirming structurally persistent or recurrent locoregional and distant disease (Table 1).

**Comparison of DTC patients with Ki-67 5–15% and ≥15%**

Among the broader cohort of 69 patients with DTC and elevated proliferation, 51 (73.9%) presented with a Ki-67 index between 5% and 15%, while 18 (26.1%) exhibited a Ki-67 index ≥ 15%. Patients in the high Ki-67 ≥ 15% group were significantly older at presentation than those in the 5–15% group (*p* = 0.002).

Definitive histopathological re-evaluation demonstrated that a Ki-67 index ≥ 15% correlated strongly with aggressive tumor biology and advanced staging. Patients with Ki-67 ≥ 15% had significantly larger tumors and higher mitotic counts (*p* = 0.002).

Histopathologically, features of local aggressiveness were markedly more common in the Ki-67 ≥ 15% subgroup, including capsule invasion (*p* = 0.006), fat-tissue invasion (*p* < 0.001), surgical-margin positivity (*p* = 0.020), lymphovascular invasion (*p* = 0.004), and perineural invasion (*p* = 0.002). Tumor necrosis was also significantly more frequent in these higher-proliferative tumors (*p* = 0.014). Consequently, patients with Ki-67 ≥ 15% presented with significantly higher Tumor-Node-Metastasis (TNM) stages (*p* = 0.025) and higher initial ATA risk classifications (*p* = 0.016).

Reflecting the extensive invasion and risk profiles identified during the clinical and staging evaluations, the definitive surgical management differed significantly between the groups. No lobectomies were performed in the Ki-67 ≥ 15% group, indicating a mandate for aggressive initial intervention (*p* = 0.004). Accordingly, lymph-node dissection was required markedly more frequently among patients with higher Ki-67 indices (*p* = 0.006).

While radioactive iodine (RAI) therapy administration rates were similar between the two groups, the necessity for additional therapeutic interventions differed sharply. The need for additional salvage surgery was markedly higher in the Ki-67 ≥ 15% group (*p* = 0.001).

During the dynamic clinical follow-up, an excellent treatment response was achieved in more than half of the Ki-67 5–15% group (58.8%), but in only 27.8% of the Ki-67 ≥ 15% group. Conversely, a structural incomplete response predominated heavily among those with a Ki-67 index ≥ 15% (*p* = 0.001). Overall, these findings underscore that a Ki-67 index ≥ 15% is strongly associated with extensive tissue invasion, higher staging, greater re-operative burden, and poorer treatment response, confirming its clinical value as an adverse prognostic marker in the broader DTC cohort with elevated proliferation. The clinical, histopathological, and follow-up characteristics of both groups are summarized in Table 2.

**Comparison of DHGTC subgroups with Ki-67 5–15% vs. ≥15%**

Among the strict sub-population of 41 patients with confirmed DHGTC, 32 (78.0%) had a Ki-67 index between 5 and 15%, and 9 (22.0%) demonstrated a Ki-67 index ≥ 15%. There were no significant differences observed between the two groups regarding sex distribution (*p* = 0.534) or median age (*p* = 0.988). The median follow-up period was also highly comparable between the cohorts (*p* = 0.816). On definitive histopathological evaluation, lymphovascular invasion was significantly more frequent among patients with Ki-67 ≥ 15% (*p* = 0.023). Other aggressive features were numerically more common in the DHGTC group with Ki-67 ≥ 15% but did not cross the threshold for statistical significance: capsule invasion (*p* = 0.194), fat-tissue invasion (*p* = 0.060), perineural invasion (*p* = 0.065), positive surgical margins (*p* = 0.297), and extracapsular extension in lymph nodes (*p* = 0.214). Initial ATA risk classification (*p* = 0.205) and TNM stage (*p* = 0.251) showed no significant differences between the groups. The definitive type of surgery performed differed significantly between these high-grade subsets (*p* = 0.014). While lobectomy was performed exclusively in patients with a lower Ki-67 index (<15%), those presenting with Ki-67 ≥ 15% more often required extensive initial surgery, including combined central and lateral neck dissections. The rate of lymph-node dissection was numerically higher in the high Ki-67 group, though this specific difference was not statistically significant (*p* = 0.114). Postoperatively, anti-Tg antibody positivity (*p* = 0.624) and thyroglobulin levels (*p* = 0.799) showed no significant differences between the groups. Similarly, no significant disparities were found regarding the administration of adjuvant RAI therapy (*p* = 0.587) or the need for additional therapeutic interventions (*p* = 0.236). At the final follow-up, patients with Ki-67 ≥ 15% displayed a numerical trend toward a higher rate of structural incomplete response, though this did not reach statistical significance (*p* = 0.123). In summary, within the strict high-grade DHGTC population, an extremely high Ki-67 index (≥15%) correlates with localized locoregional aggressiveness (lymphovascular invasion and extensive surgery). However, the lack of statistical significance regarding final therapeutic response, staging, or overall oncologic outcomes should be interpreted with caution, as it may be a reflection of limited statistical power due to small subgroup sample sizes rather than a true absence of clinical association. All the findings of DHGTC patients with Ki-67 5–15% or ≥15% are summarized in Table 3.

## 4. Discussion

The 2022 WHO update introduced DHGTC as a distinct category. This entity shows well-differentiated follicular features yet displays high-grade changes—such as necrosis or brisk mitotic activity—associated with an intermediate prognosis, setting it apart from both classic differentiated and poorly differentiated thyroid carcinoma. High-grade histologic features in thyroid cancer have traditionally been pooled with data from PDTC and, at times, even anaplastic carcinoma. Consequently, the clinical significance of identifying DHGTC as an independent category in the 2022 WHO classification remains largely unexplored. Because this entity has only recently been defined, robust prospective data, long-term outcomes, and evidence-based management guidelines are still lacking [6,7].

Our analysis focused on their preoperative clinical characteristics, postoperative histopathological features, responses to key therapeutic interventions, including surgery and radioiodine (RAI) therapy—and their dynamic risk stratification at the most recent follow-up visit. The combined prevalence of PDTC and DHGTC accounts for 1% to 6.7% of all thyroid carcinomas [8,9,10,11,12]. Since data on necrosis and mitotic count are often reported together with PDTC, the exact prevalence of high-grade features in well-differentiated carcinomas and thus of DHGTC alone remains unclear. Among the 3100 operated DTC cases in our cohort, we identified 56 cases of DHGTC and 103 cases of PDTC, representing 5.2% of the total consistent with previous reports. The male-to-female ratio was nearly equal, and the mean age was 53 years, consistent with previous reports indicating that the disease typically occurs in the fifth or sixth decade without a gender predilection [13].

On retrospective assessment of TI-RADS scores, 60% of patients with DHGTC were classified in the moderate- or high-risk categories. In contrast, nearly 40% lacked classic high-suspicion ultrasound findings, such as marked hypoechogenicity, microcalcifications, increased anteroposterior diameter, or irregular margins. Cytologically, most nodules were Bethesda V (30.4%) or VI (25%), indicating that more than half already showed highly suspicious or frankly malignant features on initial FNAB; however, over one-third fell into Bethesda III or IV (16.1% and 21.4%, respectively), while only 7.1% were Bethesda I and no Bethesda II similar to a previous report [14].

These observations suggest that although DHGTC frequently manifests clearly malignant cytology, a clinically relevant subset presents with indeterminate or even low-suspicion ultrasound patterns, underscoring the need to integrate sonographic, cytologic, and molecular information when assessing nodules that may harbor high-grade disease. Molecular testing for BRAF and RAS somatic mutations was performed using Polymerase Chain Reaction (PCR) in only four patients in the Bethesda III category and two were BRAF V600E-positive. The updated 2023 Bethesda System is valuable for thyroid nodule screening (Positive Predictive Value (PPV) 97–99%) but not fully accurate for detecting high-grade disease [15]. Certain cytologic clues like pleomorphic clusters with oncocytic/clear cytoplasm, nuclear elongation and clearing, micronucleoli, and evidence of mitoses or karyorrhexis-type necrosis may signal high-grade tumors, yet a definitive DHGTC diagnosis on cytology remains challenging [16]. No patient exhibited cytologic features predictive of high-grade disease in our study.

On histopathology, the most common DTC type was the classical variant of PTC, accounting for more than 60% of the cases, followed by Invasive Encapsulated Follicular Variant of Papillary Thyroid Carcinoma (IEFVPTC), accounting for 16%, markedly higher than previous reports [14]. In our cohort, only 5% of DHGTCs were the tall cell variant, a frequency markedly lower than that reported in a previous series, where 42% of DHGTCs exhibited tall cell morphology [16]. Before the latest WHO reclassification, most pathologists, and consequently earlier studies, grouped such tumors under conventional PTC–tall cell variant, thereby blurring the outcome data for classic PTC-TC and obscuring the true incidence and clinical behavior of DHGTC tall cell subtype. Another possible explanation for these findings is practice prior to the 2022 WHO fifth-edition update of reporting all small thyroid carcinomas simply as “microcarcinomas,” which likely led to under-recognition of tall cell variant microcarcinomas small in size but as aggressive as their microcarcinoma counterparts.

The mean mitotic count was 5.73 per 2 mm^2^, and the average Ki-67 proliferative index reached 26%—higher than previously reported [17] but still within the 10–30% range described by the WHO (1). Ki-67 data were missing in nine patients, measured <5% in six, ≥5% in 41, and exceeded 15% in only nine DHGTC cases. Necrosis was present in 87% of tumors. Additional aggressive clinicopathologic features, such as angioinvasion, lymphatic invasion, and extrathyroidal extension, were also common, consistent with previous reports [7,8]. In those reports, TERT promoter mutations were markedly more frequent in DHGTCs compared with PTCs lacking high-grade features [18,19], whereas the prevalence of BRAF mutations did not differ significantly. TERT promoter mutation data were unavailable, as only RAS and BRAF testing is routinely performed in our center.

Only 10% of patients underwent lobectomy, whereas the remaining majority had total thyroidectomy (BTT) with or without central and/or lateral lymph-node dissection. The mean tumor diameter was 5.09 ± 2.86 cm, comparable to previous reports [17,20]. The low frequency of conservative surgery likely reflects the relatively large nodule size at presentation, the high proportion of cytology results reported as malignant or suspicious for malignancy, and the presence of high-risk ultrasound features. This supports the 2025 ATA guideline recommending total thyroidectomy as a valid option even for 2–4 cm tumors without additional risk factors [2]. Lymph-node dissection, performed in 30% of cases, was indicated by extrathyroidal extension or sonographically and/or cytologically proven pathologic lymph nodes in the central compartment.

According to the 2015 ATA guidelines, which do not include the DHGTC category, 80% of patients were classified as intermediate- to high-risk, whereas by the 2025 ATA guidelines all patients were considered high-risk for recurrence. Nearly all received adjuvant-dose RAI. Structural or biochemical incomplete response was observed in 42% of cases, and 28% required additional therapy after the initial RAI. The most common secondary interventions were compartment dissection and repeat RAI for locoregional recurrence; additionally, two patients underwent radiotherapy and two received MKI treatment. These outcomes are consistent with the approximately 30% recurrence rate reported in the updated 2025 ATA risk stratification [2].

The prognostic value of combining Ki-67 expression, necrosis, and mitotic activity in predicting tumor aggressiveness has long been a subject of debate in the field of thyroid carcinoma [11]. Multiple studies have proposed stratifying differentiated thyroid carcinoma into low-, intermediate-, and high-risk groups based on Ki-67 labeling indices, commonly using cut-off values of <5%, 5–10%, and >10%, respectively. It was found to be an independent determinant for disease free survival (DFS), Tg doubling time and tumor volume doubling time [21,22]. In this study, among 3100 patients, we identified 69 cases with a Ki-67 index greater than 5% and stratified them into two groups—Ki-67 <15% and ≥15%—irrespective of histologic subtype. This cohort included both DHGTC and non-DHGTC cases.

In our broader DTC cohort, a Ki-67 index ≥ 15% clearly delineated a highly aggressive phenotype characterized by advanced patient age, larger tumor volumes, extensive locoregional invasion, and a baseline risk that translated into significantly poorer dynamic treatment responses. This aligns with the established literature positioning Ki-67 as a robust adverse marker in general differentiated thyroid cancers. However, when we isolated the strict DHGTC population to observe the impact of this extreme proliferation within an already high-grade tumor environment, the biomarker’s behavior shifted. Notably, within the strict high-grade DHGTC subgroup, a Ki-67 index ≥ 15% was significantly associated only with lymphovascular invasion and more extensive surgery, whereas final therapeutic responses and staging did not reach statistical significance.

However, this finding must be interpreted with caution. Given the small sample size of the DHGTC subgroup with Ki-67 ≥ 15% (*n* = 9), this lack of statistical significance may simply be a reflection of limited statistical power rather than a true absence of additional prognostic value. Larger, multicenter cohorts are required to definitively determine whether extremely high proliferative activity further stratifies outcomes within already high-grade thyroid malignancies. From a molecular and tumor biology perspective, the apparent lack of additional prognostic stratification by an extremely high Ki-67 threshold (≥15%) within an already established high-grade cohort may be explained by a biological ‘phenotypic ceiling effect.’ Once a differentiated thyroid carcinoma status progresses to a definitive high-grade category—driven by its core defining features of tumor necrosis or an elevated mitotic count (>5 per 2 mm^2^)—the critical intracellular pathways governing tumor aggressiveness, locoregional invasion, and early recurrence are already fully and maximally activated [23,24]. In this specific high-grade microenvironment, an incremental shift in the Ki-67 proliferation index from an intermediate-elevated state (5–15%) to an extreme state (≥15%) reflects hyperproliferative cellular kinetics that may not linearly translate into further accelerated clinical progression or distinct radioactive iodine resistance patterns. Furthermore, the clinical behavior of DHGTC is heavily dictated by co-existing and cumulative driver molecular alterations, such as *TERT* promoter mutations, *TP53* co-mutations, or complex somatic copy number variations. These genetic alterations independently govern long-term oncologic outcomes and treatment failure, thereby potentially overshadowing the incremental prognostic utility of localized, translation-level cellular proliferation markers like Ki-67.

This study has several limitations. First, it is a retrospective analysis with a relatively short follow-up period and a relatively small sample of DHGTC patients (*n* = 56), particularly within the high Ki-67 tier (*n* = 9), which significantly limits the statistical power to detect smaller but potentially clinically meaningful differences between subgroups. Importantly, comprehensive molecular characterization was unavailable; TERT promoter mutations were not evaluated, as our institution routinely performs only BRAF and RAS somatic mutation testing. Furthermore, since molecular analysis was performed in only a very limited subset of four patients with Bethesda III cytology, no broad conclusions regarding the genetic profile of DHGTC can be drawn from our data, representing a major limitation of this study. In addition, for patients operated on before 2022, the diagnosis of DHGTC was established through a retrospective report-based reclassification by a specialist endocrine pathologist via a comprehensive pathology database search. While original physical glass slides were not systematically re-reviewed for every historical case, our institution utilizes standardized synoptic reporting where mitotic counts (per mm^2^) and necrosis are mandatory components, ensuring high diagnostic reliability. Furthermore, any historical cases with ambiguous or missing data regarding these criteria were strictly excluded to preserve cohort integrity.

## 5. Conclusions

The present study provides one of the most comprehensive real-world characterizations of DHGTC since its recognition in the 2022 WHO classification. By disentangling these tumors from the historical overlap with poorly differentiated and anaplastic thyroid carcinoma, we demonstrate that DHGTC carries a clinically relevant burden of aggressive histopathologic features—high mitotic activity, frequent necrosis, and a Ki-67 index that often exceeds conventional cut-offs—while maintaining well-differentiated architecture. We confirm that a higher Ki-67 index is associated with larger tumor size, more extensive locoregional invasion, and a greater need for additional therapy. Yet, once high-grade differentiation is established, the additional prognostic value of a Ki-67 index above 15% remains inconclusive within this cohort, as potential differences may be obscured by the limited statistical power resulting from our small subgroup size.

## Figures and Tables

**Table 1:** Demographic, clinical, histopathological, and follow-up characteristics of the DHGTC group.

Parameter	Value	*n* (%)
**Total number of patients (*n*)**	56	
**Demographic and Clinical Features**		
Age (years), mean ± SD	54.09 ± 14.64	
Sex Female	29	51.8
Tumor size (cm), mean ± SD	5.09 ± 2.86	
**Surgical Management**		
BTT	50	89.3
Lobectomy	6	10.7
LN Dissection	17	30.4
**Histopathological Characteristics**		
Papillary Thyroid Carcinoma (PTC) Variants	53	94.6
Follicular/Oncocytic Carcinoma (FTC/OTC)	3	5.4
Size of the lymph node metastasis (mm), mean ± SD	12.8 ± 0.4	
Capsule invasion	38	67.9
Fat tissue infiltration	11	19.6
Surgical margins (tumor positive)	22	39.3
Lymphovascular invasion	29	51.8
Perineural invasion	7	12.5
Extracapsular extension in LN *	4/17	23.5
Necrosis	49	87.5
Number of cases with mitosis (≥5/2 mm^2^)	11	19.6
**Risk Stratification and Treatment**		
**ATA 2015 Initial Risk Score**		
Low	11	19.6
Intermediate	31	55.4
High	14	25
RAI treatment	51	91.1
Additional therapy requirement	16	28.6
**Follow-up and Response to Therapy**		
Post-op Tg median (min–max), ng/mL	1.2 (0.2–30,000)	
Anti-Tg positivity after operation (positive)	14	25
Dynamic Risk Score at Last Visit **		
Excellent Response	17	30.4
Indeterminate Response	13	23.2
Biochemical Incomplete Response	4	7.1
Structural Incomplete Response	20	35.7
Follow-up (months), mean ± SD	23.6 ± 13.8	

* Percentage calculated based on the subpopulation that underwent lymph node dissection (*n* = 17) to ensure statistical accuracy; ** Missing data for 2 cases (3.60%) in the dynamic risk stratification are acknowledged and excluded from the active percentage calculation as per reviewer guidelines.

**Table 2:** Comparison of Demographic and Clinicopathological Features of DTC Patients Based on Ki-67 Proliferation Index Cut-offs (5–15% vs. ≥15%).

Variable (*n* = 69)	Ki-67 > %5 But <15 (*n* = 51)	Ki-67 ≥ 15	*p* Value
		(*n* = 18)	
**Age (median)**	50.0 (21–81)	63.0 (32–78)	0.002
**Sex (Female)**	39 (76.5%)	11 (61.1%)	0.171
**Type of Surgery**			0.004
Lobectomy	7 (13.7%)	0 (0.0%)	
BTT	34 (66.7%)	11 (61.1%)	
BTT + Central	8 (15.7%)	5 (27.8%)	
BTT + Central + Lateral	2 (3.9%)	2 (11.1%)	
**Lymph Node Dissection**	10 (19.6%)	11 (61.1%)	0.006
Completion Surgery after lobectomy *	7/10	0/0	0.286
**Histopathological findings**			
Tumor size (cm), median	2.0 (0.5–10)	4.0 (1–11)	0.006
Capsule Invasion (if reported)	21 (41.2%)	14 (77.8%)	0.006
Fat Tissue Invasion (if reported)	8 (15.7%)	11 (61.1%)	<0.001
Surgical Margin Tumor Positivity	8 (15.7%)	8 (44.4%)	0.02
Lymphovascular Invasion (if reported)	20 (39.2%)	14 (77.8%)	0.004
Perineural Invasion (if reported)	4 (7.8%)	14 (77.8%)	0.002
Extracapsular Extension in LN **	7/10 (70%)	8/11 (72.7%)	0.615
Necrosis (if reported)	4/49 (8.2%)	6/15 (40%)	0.014
Number of mitosis (median)	3 (1–7)	7 (1–10)	0.002
**Initial Risk Classification**			0.016
Low	25 (49.0%)	2 (11.1%)	
Intermediate	18 (35.3%)	10 (55.6%)	
High	8 (15.7%)	6 (33.3%)	
**TNM Stage**			0.025
Stage I	30 (58.8%)	5 (27.8%)	
Stage II	9 (17.6%)	1 (5.6%)	
Stage III	3 (5.9%)	2 (11.1%)	
Stage IVa	4 (7.8%)	5 (27.8%)	
Stage IVb	5 (9.8)	5 (27.8%)	
**RAI Treatment**	42 (82.4%)	16 (88.9%)	0.196
**Need for Additional Surgery**	6 (11.8%)	10 (55.6%)	0.001
**Dynamic Risk Classification (last follow-up)**			0.001
Excellent Response	30 (58.8%)	5 (27.8%)	
Indeterminate Response	11 (21.6%)	0 (0,0%)	
Biochemical Incomplete Response	2 (3.9%)	1 (5.6%)	
Structural Incomplete Response	8 (15.7%)	12 (66.7%)	
**Final Anti-Tg**	13 (25.5%)	1 (6.7%)	0.544

* Completion surgery ratio describes patients who successfully underwent completion thyroidectomy out of those initially indicated. ** Percentage calculated based only on patients with proven lymph node metastasis who underwent node dissection (*n* = 10 for Ki-67 5–15% group; *n* = 11 for Ki-67 ≥ 15% group).

**Table 3:** Demographic, clinical, and histopathological characteristics and follow-up outcomes of DHGTC patients: A comparison of Ki-67 5–15% and ≥15% subgroups.

Variable (*n* = 41)	Ki-67 5–15%	Ki-67 ≥ 15%	*p* Value
	(*n* = 32)	(*n* = 9)	
**Sex (Female)**	16 (50.0%)	5 (55.6%)	0.534
**Age median (min–max)**	54.0 (28–81)	48.0 (32–78)	0.988
**Follow up months median (min–max)**	30.0 (6–79)	29.0 (15–61)	0.816
**Type of Surgery**			0.014
Lobectomy	6 (18.8%)	0 (0.0%)	
BTT (bilateral total thyroidectomy)	19 (59.4%)	4 (44.4%)	
BTT + central	3 (9.4%)	0 (0.0%)	
BTT + central +lateral	4 (12.5%)	5 (55.6%)	
Post-operative anti-Tg antibody (Positive)	21 (65.6.%)	6 (66.7%)	0.624
Post op Tg	3.7 (0.2–30,000)	3.3 (0.2–23,960)	0.799
Lymph-node dissection performed	11 (34.4%)	6 (66.7%)	0.114
Completion thyroidectomy performed	6/6 (100%)	0/0 (0%)	0.453
**Histopathological findings**			
Tumor Size	5.5 (2.1–8.0)	10.00 (7.1–12.0)	0.359
Capsular invasion	20 (62.50%)	8 (88.9%)	0.194
Fat tissue invasion	6 (18.8%)	4 (44.4%)	0.060
Positive surgical margin	10 (31.25%)	4 (44.45%)	0.297
Lymphovascular invasion	15 (46.9%)	8 (88.9%)	0.023
Perineural invasion	3 (9.4%)	4 (44.4%)	0.065
Extracapsular extension in lymph node	2/11 (18.1%)	4/6 (66.6%)	0.214
**Initial risk classification**			0.205
Low	8 (25.0%)	1 (11.12%)	
Intermediate	17 (53.12%)	5 (55.55%)	
High	6 (21.88%)	3 (33.33%)	
**TNM stage**			0.251
Stage I	9 (28.13%)	1 (11.12%)	
Stage II	4 (12.50%)	0 (0%)	
Stage III	8 (25.0%)	3 (33.33%)	
Stage Iva	7 (21.87%)	2 (22.2%)	
Stage 4b	4 (12.50%)	3 (33.33%)	
Received RAI therapy	30 (93.75%)	9 (100.0%)	0.587
Need for additional therapy	10 (31.25%)	4 (40.0%)	0.236
**Dynamic risk classification at last follow-up**			0.123
Excellent	10 (31.25%)	2 (22.22%)	
Indeterminate	9 (28.12%)	1 (11.11%)	
Biochemical incomplete	5 (15.63)	1 (11.11%)	
Structural incomplete	8 (25.0%)	5 (55.56%)	

## Data Availability

The data presented in this study are available on request from the corresponding author. The data are not publicly available due to privacy and ethical restrictions.

## Supplementary Materials

The following supporting information can be downloaded at https://www.mdpi.com/article/10.3390/jcm15114173/s1, Figure S1: Flowchart of the study population selection process.

## Abbreviations

The following abbreviations are used in this manuscript:

AJCC	American Joint Committee on Cancer
ATA	American Thyroid Association
Bethesda	The Bethesda System for Reporting Thyroid Cytopathology
BTT	Bilateral Total Thyroidectomy
CT	Computed Tomography
DHGTC	Differentiated High-Grade Thyroid Carcinoma
DTC	Differentiated Thyroid Carcinoma
FNAB	Fine-Needle Aspiration Biopsy
FTC	Follicular Thyroid Carcinoma
IEFVPTC	Invasive Encapsulated Follicular Variant of Papillary Thyroid Carcinoma
LN	Lymph Node
LND	Lymph Node Dissection
OTC	Oncocytic Thyroid Carcinoma
PCR	Polymerase Chain Reaction
PDTC	Poorly Differentiated Thyroid Carcinoma
PET	Positron Emission Tomography
PPV	Positive Predictive Value
PTC	Papillary Thyroid Carcinoma
RAI	Radioactive Iodine
ROC	Receiver Operating Characteristic
SD	Standard Deviation
SPSS	Statistical Package for the Social Sciences
Tg	Thyroglobulin
TgAb	Anti-thyroglobulin Antibody
TI-RADS	Thyroid Imaging Reporting and Data System
TNM	Tumor-Node-Metastasis
US	Ultrasonography
WHO	World Health Organization
