# Supplementary figures and images for: Re-Analyzing Differentiated High-Grade Thyroid Carcinoma and Elevated Ki-67 Proliferation: A Single-Center Retrospective Study
Source: J Clin Med. 2026 May 28;15(11):4173. doi: 10.3390/jcm15114173 (PMC13258479; doi:10.3390/jcm15114173)

**Figure S1. Flowchart of the study population selection process.**

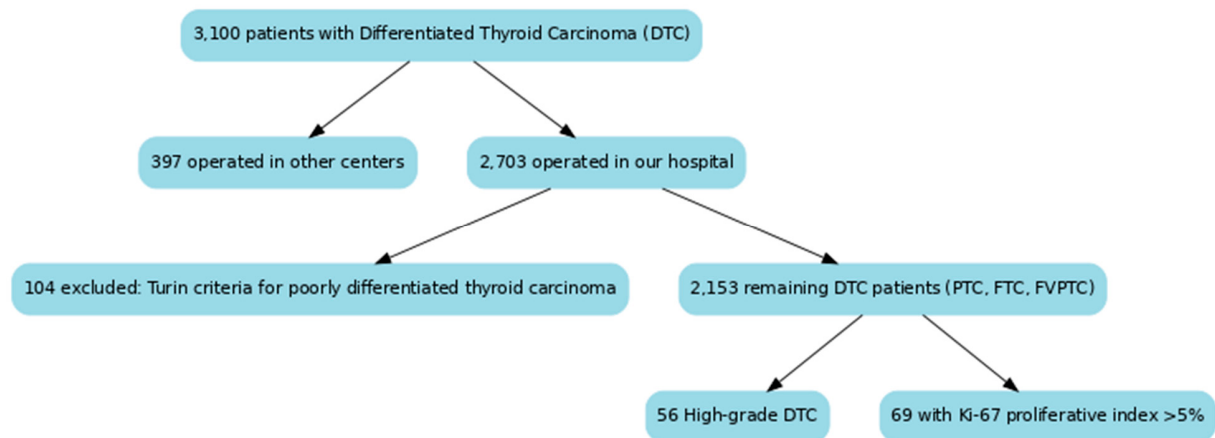

Supplement: Supplementary file 1 [file jcm-15-04173-s001.zip › jcm-4304145-supplementary.pdf]
